# A Rare Case of Pertuzumab-Induced Toxic Epidermal Necrolysis

**DOI:** 10.7759/cureus.39797

**Published:** 2023-05-31

**Authors:** Mohamed Zakee Mohamed Jiffry, Felipe Carmona Pires, Maria A Perozo, Napat Rangsipat, Daniel Tabares

**Affiliations:** 1 Internal Medicine, Danbury Hospital, Danbury, USA; 2 School of Medicine, American University of the Caribbean, Cupecoy, SXM

**Keywords:** anti-her2 therapy, autoimmune blistering skin disease, toxic epidermal necrolysis (ten), pertuzumab, her-2 positive breast cancer

## Abstract

Pertuzumab is a targeted therapy drug that is employed in the management of human epidermal growth factor receptor 2 (HER2)-positive breast cancer and works by blocking the ability of cancer cells to receive growth and proliferation signals. Toxic epidermal necrolysis (TEN) is a severe cutaneous manifestation characterized by widespread erythema, necrosis, and bullous detachment of the skin involving more than 10% of the body surface area (BSA) and may be precipitated by an immunologic response to the administration of certain medications. However, TEN development as a consequence of HER2 inhibitor therapy has not been described in the existing literature. A 44-year-old female with a history of metastatic breast cancer to the liver presented with a diffuse blistering rash following a first-time administration of pertuzumab three days prior. Her rash began as painful and pruritic blisters 12 hours after the last infusion of pertuzumab and progressed to involve her arms, chest, groin, and thighs with a positive Nikolsky sign. She was managed supportively with high-dose steroids and antihistamines, and although her hospital course was complicated by hypotension requiring pressor support, she gradually made a full recovery and was released to a rehabilitation facility.

## Introduction

Pertuzumab (Perjeta) is a targeted therapy that inhibits human epidermal growth factor receptor-2 (HER2), a protein found in HER2-positive breast cancers. It works by blocking the cancer cells' ability to receive growth signals, making it an important treatment option for HER2-positive breast cancer [1].

Toxic epidermal necrolysis (TEN) is a severe skin reaction caused by certain drugs, including antibiotics, non-steroidal anti-inflammatory drugs (NSAIDs) [2], antiretroviral drugs, and others. It is characterized by erythema, necrosis, and bullous detachment of the skin and mucosal membranes. The exact cause of TEN is not fully understood, but it is believed to involve immunologic reactions, reactive metabolites from drugs, and genetic factors [3,4]. To the best of our knowledge, no case reports exist in the current literature detailing TEN after administration of the HER2 inhibitor class of therapies.

We present a 44-year-old female with a history of HER2-positive left breast cancer and liver metastases who developed TEN after her first dose of pertuzumab.

## Case presentation

A 44-year-old female presented to the emergency department with a diffuse blistering rash following a first-time administration of pertuzumab three days prior. Her medical history was significant for triple-negative right breast cancer with biopsy-proven liver metastases and estrogen receptor (ER) positive/progesterone receptor (PR) negative/HER-2 positive left breast cancer.

Her home medications included trazodone, oxycodone, and ondansetron. Her initial chemotherapy regimen, which began two months prior to presentation, consisted of paclitaxel and trastuzumab. She received a total of three doses of paclitaxel and one dose of trastuzumab during that time without any dermatologic side effects. Three days prior to presentation, the patient received a fourth dose of paclitaxel, a second dose of trastuzumab, and pertuzumab for the first time. The patient denied any known drug allergies, use of herbal supplements, or any changes to her home medication regimen.

Her rash began 12 hours after her last chemotherapy infusion and started as painful, pruritic blisters overlying her upper back and neck. Over the next 24 hours, the rash spread to the arms, chest, abdomen, groin, and thighs. The blisters progressively grew in size, and some of them burst spontaneously, releasing serosanguineous fluid.

**Figure 1:**
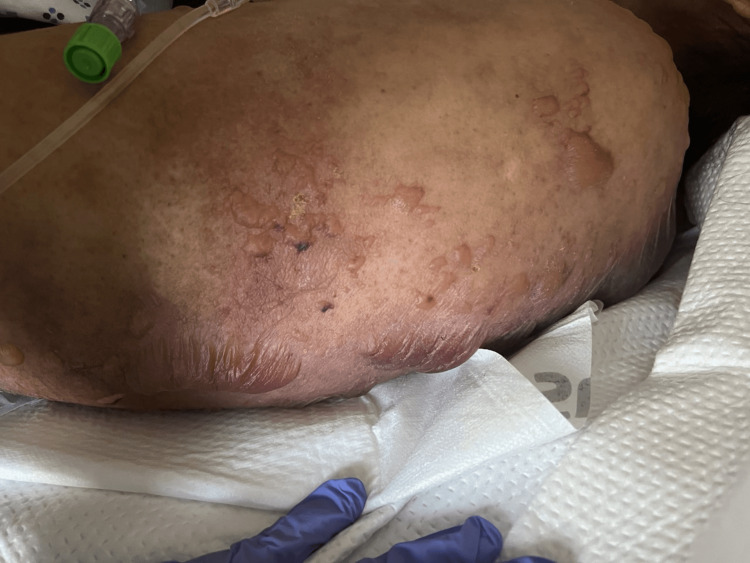
Skin findings over upper back showing areas of bullae formation, blistering and erythema characteristic of toxic epidermal necrolysis

The patient presented to the ER with normal vital signs except for a heart rate of 114/min. A physical exam revealed a well-developed female in visible distress. A skin exam demonstrated multiple vesicles and large bullae surrounded by erythema (Figure 1).

There were areas of denuded skin. Nikolsky's sign was positive. The estimated body surface area (BSA) involved was 35%. Oral lesions were not observed.

A complete blood count was significant for leukocytosis of 22,900/µL (reference range: 4,500-11,000/µL), thrombocytopenia of 100,000/µL (reference range: 150,000-400,000/µL), hemoglobin of 9.1 g/dL (reference range: 12-16 g/dL). Renal and liver function tests were normal. CRP was 51.3 mg/L (reference range: <10.0 mg/L).

A skin biopsy revealed epidermal detachment with superficial epidermal necrosis and dyskeratotic keratinocytes consistent with TEN. She was initially transferred to a critical care unit and started on IV steroids with dexamethasone (10 mg twice daily) and diphenhydramine (25 mg every eight hours). Topical bacitracin was applied over the skin for infection prophylaxis, and wound care with sterile saline and Xeroform gauze was applied. IV hydration to reconstitute volume loss was ongoing.

Her course in the critical care unit was further complicated by hypotension, with the differential diagnosis including septic versus hypovolemic shock, for which she was started on IV vasopressors, phenylephrine, and norepinephrine to maintain a mean arterial pressure greater than 65 mmHg. She was subsequently transferred to a dedicated burn unit for further management. She continued to improve with respect to TEN and was released to a physical rehabilitation facility.

## Discussion

Stevens-Johnson syndrome (SJS) and TEN are severe hypersensitivity reactions with mucocutaneous involvement and an average mortality rate of 1-5% in SJS and 25-35% in TEN [5]. Patients in both conditions present with skin erythema, tenderness, flat atypical target lesions, blisters, hemorrhagic erosions, and epidermal detachment. Pressure applied to the skin causes epidermal detachment (Nikolsky sign), though it is not specific to only SJS/TEN. SJS and TEN differ in the percentage of skin involvement, with SJS involving less than 10% of the total body surface, whereas TEN involves more than 30% of the total body surface area. Involvement between 10% and 30% is considered SJS-TEN overlap.

These hypersensitivity reactions are rare, with the mean estimated incidences of SJS, SJS/TEN overlap, and TEN of 9.2, 1.6, and 1.9 per million adults per year in the United States, respectively [6]. Several known drugs to induce SJS and TEN include allopurinol, trimethoprim-sulfamethoxazole, other sulfonamide antibiotics, aminopenicillins, cephalosporins, quinolones, carbamazepine (CBZ), phenytoin, phenobarbital, and NSAIDs of the oxicam type [5]. Certain infections, such as Mycoplasma pneumoniae and the herpes simplex virus, can also induce this reaction. A literature review between 1968 and 2012 of the Food and Drug Administration Adverse Event Reporting System (FAERS) reported significant signals of SJS and TEN to some anticancer drugs, including bendamustine, busulfan, chlorambucil, fludarabine, lomustine, and procarbazine [7,8]. How different medications and infections can lead to SJS and TEN is unclear, but cytotoxic T lymphocytes (CD8+), FasL, and granulysin are believed to be responsible for disseminated keratinocyte apoptosis in SJS/TEN [5].

Our patient had TEN involving approximately 35% of her body surface area following her first exposure to pertuzumab, a monoclonal antibody acting as an inhibitor of HER2 dimerization, for her treatment of metastatic breast cancer. Pertuzumab is used in combination with trastuzumab and docetaxel for the treatment of HER2+ metastatic breast cancer. Pertuzumab is generally well tolerated, with some side effects such as diarrhea, nausea, vomiting, asthenia, and a rash. Skin rashes, as reported in most clinical trials, are low-grade (grade 1 or 2) with a papulopustular (acneiform) phenotype [1]. The study also reported that tumor types seem to influence the risk of rash incidence in patients who are on pertuzumab, with breast, ovarian, fallopian tube, and peritoneal cancer having a higher incidence of rashes than prostate cancer, and the overall incidence of rash with pertuzumab in breast cancer was 28.5% (95% CI 25.5-31.7%).

An important consideration to make in this particular case is the clear temporal association between the administration of pertuzumab and the development of our patient's symptoms, as most cases of TEN induced by an offending medication have been studied to have a median latency time between the beginning of use and the onset of TEN of less than four weeks [9].

Genetic predisposition has been shown to link SJS/TEN with certain human leukocyte antigens (HLA) and drugs. CBZ-induced SJS/TEN is linked with HLA-B*15:02 in Asian populations, including those in China, Thailand, Malaysia, and India, though there is no association with HLA-B*15:02 in Japanese, Korean, or European populations [10]. On the other hand, CBZ-induced SJS/TEN in Japanese, Korean, and European populations is linked to HLA-A*31:01. HLA association is therefore not a sole factor in the development of SJS/TEN but seems to differ among different ethnicities as well. Other drugs like allopurinol are linked to HLA-B*58:01 in Taiwanese, Japanese, Korean, Thai, and European individuals, while abacavir is associated with HLA-B*57:01. Screening guidelines have been implemented to prevent SJS/TEN development in some medications [11]. Given that our patient is of Indian heritage, a further look into the relationship between HLA association and the development of SJS/TEN in different cancer drugs may be beneficial for future research.

Management of TEN/SJS mainly consists of immediate withdrawal of suspected drugs and supportive care. Maintaining electrolyte balance and proper wound care are important aspects of management. Early use of corticosteroids within 24-48 hours of symptom onset has been shown to have a good outcome, as has the use of other immunomodulators such as IVIG, cyclosporine, tacrolimus, and cyclophosphamide [12]. The survival rate is shown to be significantly higher in patients who were transferred to the burn unit within seven days after disease onset [5].

## Conclusions

This case demonstrates the potential link between toxic epidermal necrolysis and the chemotherapy drug pertuzumab. Although rare, toxic epidermal necrolysis can be life-threatening and requires prompt diagnosis and management. Early withdrawal of suspected drugs, supportive care, and the use of corticosteroids and other immunomodulators can help manage this condition. Further research on the relationship between human leukocyte antigen association and the development of toxic epidermal necrolysis with different cancer drugs may be beneficial in the future, particularly for patients of different ethnicities.
